# Surgery

**Published:** 1890-08

**Authors:** John Parmenter


### Progress in Medical Science.

### SURGERY.

By JOHN PARMENTER, M. D.

### PENETRATING GUN-SHOT WOUNDS OF THE ABDOMEN.

DR. AUGUST SCHACHNER, in the *Annals of Surgery* for June, 1890, contributes an article entitled Penetrating Gun-shot Wounds of the Abdomen, in which he gives in detail the results of some experiments upon dogs in this line of surgery. The experiments were divided into three classes: gun-shot wounds with interference, without interference, and of separate organs.

The first class, comprising thirty-two experiments, gave seventeen recoveries, 45 per cent.; (one experiment in which death followed immediately is omitted). In all except four of the remaining fourteen, terminating fatally, death occurred during or at the end of the first twenty-four hours after operation. Death followed in one and a half, three, three and a half, and four and a half days respectively in the four above excepted. The cause of death was as follows: Two from hemorrhage, 14.2 per cent.; five from shock primarily, 35.7 per cent.; six from sepsis, 48.2 per cent.; and one from shock consecutive to an operation for obstruction.

The second class consisted of five. These went without operation. One recovered. Death resulted in one from shock; in two from hemorrhage; in one from sepsis. The author thinks the recovery of one animal due to the probable slight injury.

The third class includes four resections, after Wölfler's method, a partial resection of the liver, a partial resection of the spleen, and one experiment upon the peritoneum. All recovered. Lastly a final experiment with Senn's test. As a result of his experiments, the author makes the following deductions:

1. In view of the uncertainties which attend these injuries, exploratory laparatomy should in every case be boldly but carefully performed, the operator being in readiness to meet any indication that the exigency of the case may demand.

2. Laparatomy in the linea alba is preférable to one performed in the course of the ball, unless there are reasons to believe that the ball became arrested short of the peritoneum, or its track infected, in which case incision and drainage should be employed.

3. Considering the objections against Senn's test as a diagnostic means of determining the necessity of a laparatomy, the possible harm outweighs to such an extent the possible benefit, that its general adoption is hardly justifiable.

4. The value of Senn's method in determining at the close of the operation the security of the intestinal tract is questionable and still *sub judice.*

5. Large intestinal wounds, not involving the mesenteric border, are best treated by partial resections.

6. Intestinal wounds upon the mesenteric border, unless very small, require a complete resection.

7. Where several large wounds are situated very close together, a single resection, including them all, should be considered.

8. Partial resections of the liver, spleen, or pancreas are feasible steps, and may be required.

9. Suturing of both openings in wounds of the liver and spleen for the arrest of hemorrhage is advisable.

10. Excepting superficial lesions, nephrectomy is the only procedure in wounds of the kidney.

11. Should obscure symptoms arise pointing to an early peritonitis, the use of salines is indicated.

12. If suppurative peritonitis is established, early exploratory incision, drainage and disinfection of the peritoneum should be undertaken.

### CEREBRAL LOCALIZATION.

In the *American Journal of Medical Sciences* for July, 1890, Prof. Andrew H. Smith relates a case in which cerebral localization was illustrated by the effect of mental impression. A gentleman consulted him for a condition of the right hand and wrist resembling writer's cramp, but differing from it in that the condition was one of tremor rather than spasm. It did not result from special or excessive use of the hand. There was also slight paresis of the right leg. Occipital pain was present, due to defective action of the ocular muscles, but the use of prisms relieved the pain and improved the condition of the hand and leg. Inasmuch as he had been treated by many physicians without avail, Prof. Smith advised him to be contented with the relief he had obtained, and gave him active employment to divert his mind. He got on very well, general health improved, hand and leg better, but still uses left hand in writing. A few days ago, in reading an article in *Harper's Magazine* for May, in which the writer describes the physical distress of signing 12,500 United States bonds in sixty-four hours, Prof. Smith's patient began to experience a severe pain, which commenced in the right hand, running up the arm into the neck, then into the occiput, where it became intense in a circumscribed spot on the left side of the head. The spot corresponded exactly to the situation of the postero-parietal convolution, the center for the wrist and hand of the opposite side, according to Ferrier. The patient knew nothing of cerebral localization, except that the brain on one side actuates the muscles on the other, and therefore the pain he complained of could not have been due to suggestion. Prof. Smith, therefore, concludes that:

> The phenomena in the hand and leg had back of them a morbid condition of the cortical center, and that this morbid condition was in such wise influenced by the direction of the mind toward the function of that center as to express itself in pain. Probably the intermediate link between the mental action and the production of pain was a vaso-motor disturbance.

He thinks that while functional activity of a cortical center may give rise to an appreciable increase of local temperature, this case would seem to show that merely occupying the imagination with a function may excite a degree of disturbance in the center corresponding to that function. The pain originating so peculiarly confirms the cortical origin of the symptoms. The implication of the leg suggests that the posterior portion of the convolution was also involved. Accepting Ferrier's earlier conclusions regarding the function of the angular gyrus in relation to ocular movements, the proximity of this gyrus to the postero-parietal convolution would probably explain the improvement in the hand and leg when coincident with that of the eyes. The author concludes by saying that "the more recent views, however, as to the location of the center for ocular movements remove it so far from this region that this explanation scarcely seems tenable." (Should

Ferrier's conclusions regarding the angular gyrus prove true after all, this case would be a most interesting one, and a few more of a similar nature would give us strong corroborative clinical evidence to aid us in localizing cerebral centers.— P.)

### A UNIQUE CASE OF APHASIA.

During the XIXth Congress of the German Surgical Society, Rosenberger, of Würzburg, described a unique case of aphasia with loss of power of communicating thought by pantomime or gesture (amimia). The case was as follows :

A boy, six years of age, sustained a kick from a horse over the left parietal bone, in consequence of which he was unconscious for twelve days, and after this time aphasic. This condition had existed five weeks when Rosenberger first saw him. At this time he had no fever and a normal pulse. Over the left parietal bone there existed three ulcers, the undermost of which was two c.m. over the left ear. It was about three c.m long, equally wide, and showed marked bulging outward and distinct cerebral pulsation. In the neighborhood of the ulcers depression of the cranial vault could be felt. A slight paralysis of the fingers of the right hand was present. The patient had a good appetite and slept well. The eyes and ears were normal. The aphasia was complete. His intelligence was unaffected, for he understood everything correctly, recognized everything, and could select what he wanted out of different other things, but he could not make himself understood by nodding or shaking his head. As soon, however, as someone showed him how to do it he could immediately imitate the movements.

Rosenberger decided to operate, for two reasons ; to remove pressure caused by the depressed bone, and to cover the prolapsed portion of brain after the plastic method of Von Bergman.

The depressed portions of bone were together six and a half c.m. long by four c.m. wide. The corresponding defect was covered with two flaps taken from the surrounding region.

The subsequent course was without fever, and the result brilliant. On the fourth day the paralysis of the hand improved, on the fifth day he moved his head voluntarily for the first time, and on the sixth he uttered a sound like the letter i. He was discharged recovered on the twenty-second day, with speech completely restored.— *Centralblatt für Chirurg.* 1890. No. 25.

